# GPX7 Facilitates BMSCs Osteoblastogenesis via ER Stress and mTOR Pathway

**DOI:** 10.1111/jcmm.16974

**Published:** 2021-10-09

**Authors:** Xuchen Hu, Boer Li, Fanzi Wu, Xiaoyu Liu, Mengyu Liu, Chenglin Wang, Yu Shi, Ling Ye

**Affiliations:** ^1^ State Key Laboratory of Oral Diseases National Clinical Research Center for Oral Diseases West China Hospital of Stomatology Sichuan University Chengdu China; ^2^ Department of Endodontics West China Hospital of Stomatology Sichuan University Chengdu China

**Keywords:** BMSCs, ER stress, GPX7, osteogenic differentiation

## Abstract

Emerging evidence indicates extensive oxidative stress is a consequence of obesity which impairs bone formation. Glutathione peroxidase 7 (GPX7) is a conserved endoplasmic reticulum (ER) retention protein, lacking of which causes accumulation of reactive oxygen species (ROS) and promotes adipogenesis. Since the imbalance between osteogenic and adipogenic differentiation of bone marrow mesenchymal stem cell (BMSC) leads to severe bone diseases such as osteoporosis, it is critical to investigate the potential protective role of *Gpx7* in osteogenesis. Here, we provide evidence that deficiency of *Gpx7* reduces osteogenesis, but increases adipogenesis in both human BMSCs (hBMSCs) and mouse mesenchymal stem cell line. Interestingly, further studies indicate this defect can be alleviated by the ER stress antagonist, but not the ROS inhibitor, unveiling an unexpected finding that, unlike adipogenesis, lacking of *Gpx7* inhibits osteogenesis mediating by induced ER stress instead of enhanced ROS. Furthermore, the mTOR signalling pathway is found down‐regulation during osteogenic differentiation in *Gpx7*‐deficient condition, which can be rescued by relief of ER stress. Taken together, for the first time we identify a novel function of *Gpx7* in BMSCs’ osteogenic differentiation and indicate that *Gpx7* may protect against osteoporotic deficits in humans through ER stress and mTOR pathway interplay.

## 1 INTRODUCTION

Obesity is an increasing global epidemic that severely affects human health and is also closely associated with bone diseases such as osteoporosis.^1^, ^2^ Extensive ER stress and oxidative stress (OS) are outcomes of obesity that contribute to adipokine accumulation, which causes aberrant lineage allocation of BMSCs and aggravates bone mass loss.^3^, ^4^, ^5^, ^6^

GPX7, also known as NPGPx, is a highly conserved member of the glutathione peroxidase (GPX) family which serves as antioxidant enzymes working with superoxide dismutase (SOD) to convert superoxide to water.^7^ In contrast with other GPXs, GPX7 lacks GPX enzymatic activity due to the absence of a critical selenocysteine residue and is primarily located in the ER.^8^ Previous studies have identified it as a novel OS sensor/transducer that transmits ROS signals to effectors by shuttling disulphide bonds and functionally alleviates stress caused by accumulated misfolded proteins in the ER.^9^, ^10^ For example, GPX7 over‐expression is demonstrated to protect the endothelium from ER OS and inflammation.^11^ Moreover, the knockdown of GPX7 has been shown to sensitize breast cancer cells to OS‐induced cell death,^12^ suggesting its importance in relieving OS. Furthermore, the expression of GPX7 is decreased during adipogenic differentiation, and the deficiency in *Gpx7* promotes pre‐adipocytes to differentiate into adipocytes via ROS‐dependent dimerization of protein kinase A regulatory subunits and activation of CCAAT/enhancer‐binding protein beta (C/EBP β).^13^ Since the commitment of BMSCs differentiation must be precisely controlled to maintain bone homeostasis, it is worth investigating whether the lack of *Gpx7* impedes osteoblastogenesis. Furthermore, the existing sequencing data show that the expression of *Gpx7* is increased in osteogenesis, suggesting that it plays a positive role in bone formation.^14^ However, the regulatory mechanism of GPX7 in osteogenesis of BMSCs remains largely unexplored.

Among the regulatory machineries of BMSCs differentiation, a proper level of ER stress plays an indispensable role in osteogenesis.^15^ Due to the need to synthesize and secret a large number of proteins for osteogenesis, a low level of ER stress occurs during osteogenic differentiation,^16^ However, excessive ER stress can suppress bone formation and even cause cell apoptosis.^17^, ^18^ ‘ER stress’ refers to a defence system that can bring the folding capacity and unfolded protein burden of ER into line and return it to its normal physiological state. When cells respond to various stresses such as over‐nutrition,^19^ obesity,^20^ type 2 diabetes^21^ and other related metabolic disorders,^22^ it can lead to the accumulation of misfolded proteins in ER, causing ER stress. To maintain ER homeostasis and alleviate ER stress, cells initiate an ER‐specific unfolded protein response (UPR). The core component of unfolded protein response is a triad of stress‐sensing proteins: inositol‐requiring enzyme 1 (IRE1), protein kinase R‐like endoplasmic reticulum kinase (PERK) and activating transcription factor 6 (ATF6).^23^ OS and ER stress are interconnected such that misfolded proteins induce ROS production; likewise, OS disturbs the ER redox state, thereby disrupting proper disulphide bond formation and protein folding.^24^ Therefore, whether ER stress and ROS accumulation mediate *Gpx7* deficiency‐decreased osteogenesis needs to be illuminated.

The interplay between ER stress and mammalian/mechanistic target of rapamycin (mTOR) has been shown to be complicated.^25^, ^26^ Nonetheless, it has been reported both ROS accumulation and UPR partially function through the mTOR pathway. The mTOR complex (mTORC), a serine/threonine protein kinase that integrates the inputs from nutrients and growth factors to control many fundamental cellular processes through two distinct protein complexes, mTORC1 and mTORC2, which containing the unique subunit Raptor and Rictor, respectively.^27^ mTORC1 functions mainly through the phosphorylation of p70 S6 kinase (P70S6K1) and the eukaryotic translation initiation factor 4E‐binding protein 1 (4EBP1), whereas mTORC2 controls the proliferation and differentiation through a distinct group of downstream targets, such as members of the AGC family of kinases (AKT). Recent genetic studies in mice have established that the mTOR pathway plays an important role in regulating multiple aspects of skeletal development and homeostasis.^28^, ^29^ *In vitro* studies also demonstrate that inhibition of mTORC1 signalling by rapamycin impairs both proliferation and osteogenic differentiation of mouse BMSCs.^30^ Like mTORC1, mTORC2 is implicated in regulating osteoblastic differentiation and function. BMSCs lacking the *Rictor* gene exhibit reduced osteogenic potential but increased capacity to undergo adipogenic differentiation *in vitro*.^31^ These findings raise the question of whether *Gpx7* deficiency causes osteogenic defects via the mTOR pathway.

Therefore, in the present study, we investigated whether *Gpx7* expression was indispensable to the osteogenic differentiation of BMSCs. We found that *Gpx7* was significantly up‐regulated during osteogenesis in hBMSCs and the M2‐10B4 bone marrow stromal cell line, and we also detected strong expression thereof during bone development and fracture healing. Moreover, *Gpx7*‐deficient hBMSCs and M2‐10B4 cells showed reduced osteogenic differentiation *in vitro*, which was alleviated by ER stress inhibitor 4‐phenylbutyric acid (4‐PBA). Importantly, *in vivo* experiments demonstrated *Gpx7*‐deficient also impaired new bone formation in the cranial defect model which could be rescued by alleviating ER stress. Mechanistically, we found that mTOR signalling functionally involved in *Gpx7*‐defective osteogenic differentiation which indicated that *Gpx7* played a critical role in mediating osteogenesis by regulating ER stress and the mTOR pathway.

## 2 MATERIALS AND METHODS

### 2.1 *In vitro* cell cultures and alkaline phosphatase (ALP), Alizarin Red staining

The M2‐10B4 cell line was purchased from ATCC (ATCC, VA, USA). hBMSCs and mouse bone marrow mesenchymal stem cells (mBMSCs) were purchased from Cyagen (HUXMA‐01001, MUCMX‐01001, Cyagen, CA, USA). M2‐10B4 was cultured in RPMI1640 medium (Gibco, Life Technologies, CA, USA), and hBMSCs and mBMSCs were in alpha modified Eagle's medium (Gibco, Life Technologies, CA, USA). All media were supplemented with 10% FBS (Invitrogen, CA, USA) and 1% penicillin‐streptomycin (Gibco, Life Technologies, CA, USA). All osteogenesis experiments were based on the treatment of odontogenic medium (OM) containing Dulbecco's modified eagle medium (Gibco, Life Technologies, CA, USA) with 10% FBS, 1% P/S, 10 mmol/L β‐glycerophosphate (Sigma‐Aldrich, St. Louis, MO, USA) and 50 µg/mL ascorbic acid (Sigma‐Aldrich, St. Louis, MO, USA). The medium was changed every 2–3 days. To assess the effect of N‐acetyl‐L‐cysteine (NAC, Sigma‐Aldrich, St Louis, MO, USA) or 4‐PBA (Sigma‐Aldrich, St Louis, MO, USA) on osteoblast differentiation, these reagents were added at the first 1 days after the transfection, respectively, then changed to OM.

ALP staining (Beyotime, Shanghai, China) was performed 4 days after treating with OM. Alizarin red staining (Solarbio, Beijing, China) according to the manufacturer's instruction was performed 7 days after OM treatment for hBMSCs and 14 days after OM treatment for M2‐10B4 cells. The density of ALP staining was measured by Image‐Pro Plus 6.0.

### 2.2 Immunohistochemical (IHC) Staining

For IHC staining, the femurs and tibias from male C57BL/6 mice were decalcified in 10% ethylenediaminetetraacetic acid (EDTA) after fixation and sectioned at 7 µm thickness. The paraffin sections were dewaxed in xylene, rehydrated with distilled water and then subjected to antigen retrieval. Tissue sections were stained based on the standard protocol for the Anti‐rabbit HRP‐DAB Kit (R&D Systems, MN, USA). The primary antibodies are anti‐OSX (Abcam, ab22552; 1:200) or anti‐GPX7 (Proteintech, 13501–1‐AP; 1:25). The cell nuclei were also labelled with haematoxylin. A microscope imaging system (Olympus BX53) was used for imaging.

### 2.3 Animal surgeries

All mouse experiments were conducted following approved protocols by the Institutional Animal Care and Use Committees of Sichuan University. Briefly, male C57BL/6 mice (8 to 10 weeks of age) were anaesthetized and the intramuscular septum was separated by blunt dissection to locate the femurs. Then, a Kirschner wire was inserted through the patellar tendon into the femoral marrow cavity. The middle diaphysis was fractured with a blade, and the wound was closed with sutures. The femurs were collected at two weeks after the fracture, fixed with 4% paraformaldehyde for 24 hrs, decalcified in 10% EDTA for 21–28 days and embedded with paraffin.

For calvarial defects, after disinfection of the skull skin and exposure of the operative area, 3.0 mm diameter defects were created in 8‐week male C57BL/6 mice under sterile conditions, 10^6^ transfected with or without 2 mM 4‐PBA‐treated mBMSCs were mixed with 100 μL of Germa gel, and 10 μL of the gel cell mixture was dropped into each bone defect area and photocured to fix. The skin was then sutured with 5–0 sutures. The mice were divided into four groups of four animals each (Control, si*Gpx7*, Control‐4‐PBA and si*Gpx7*‐4‐PBA). After 4 weeks from the operation, the mice were sacrificed. The upper part of the skulls was removed and scanned using VIVA 40CT 64GB micro‐CT system (Scanco Medical AG, Bassersdorf, Switzerland) at a resolution of 15 µm and with the settings 70 kVp and 200 µA. The three‐dimensional images and the bone morphometric parameter BV/TV were constructed and analysed with μCT Ray V3.0 (Scanco Medical).

### 2.4 Statistical analysis

All assays were repeated in three independent experiments. All data were presented as mean ±SD. Significance was determined by Student's t test for pairwise comparison and one‐way ANOVA with Bonferroni post‐test for multiple comparisons using GraphPad Prism 6.0 software (Graphpad Software, Inc., La Jolla, CA, USA). The significance level was set at *p* < 0.05.

## 3 RESULTS

### 3.1 The expression of GPX7 is up‐regulated during osteogenesis

**FIGURE 1:**
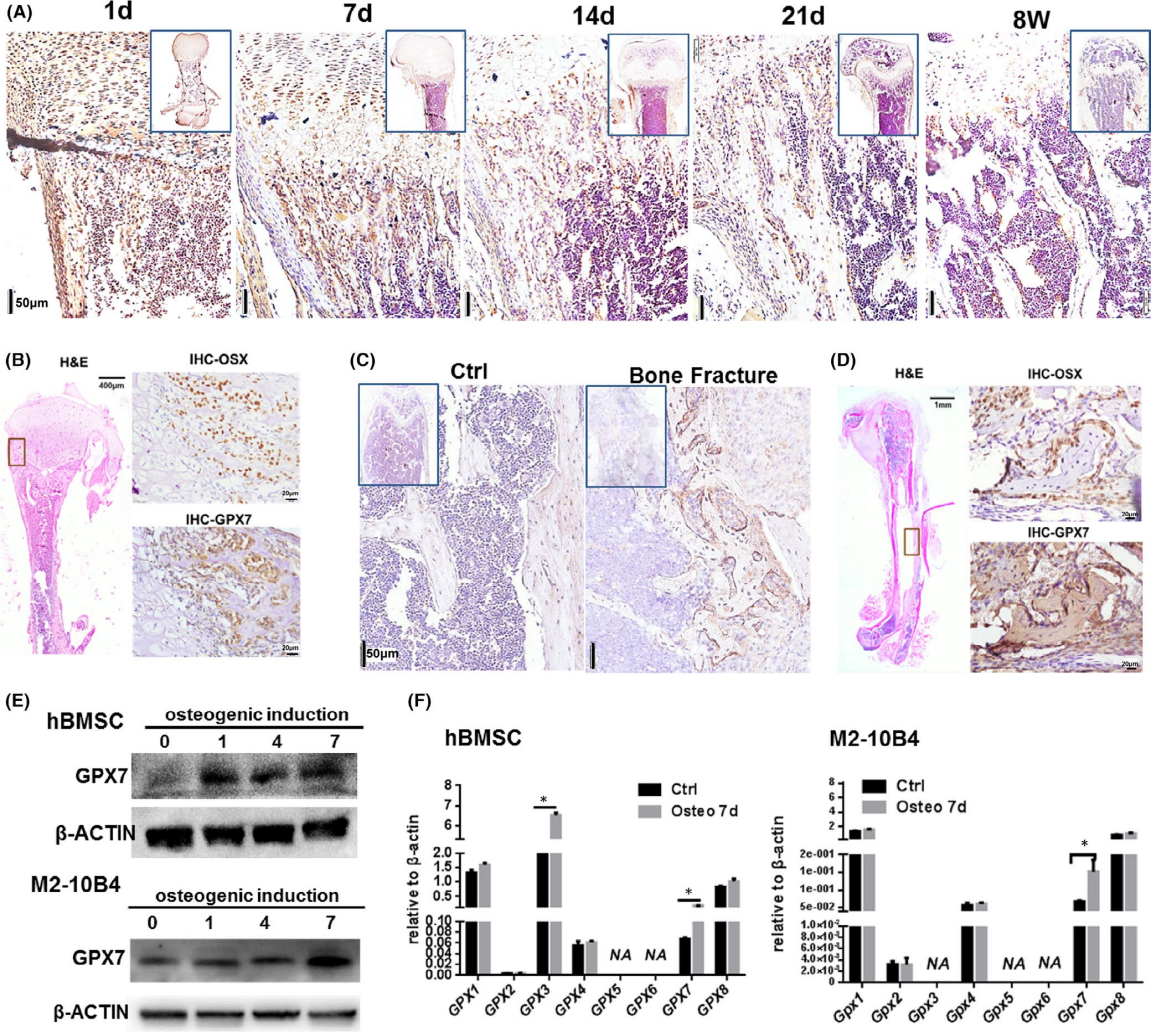
The expression of GPX7 is up‐regulated during osteogenesis. (A) IHC staining showing the distribution of GPX7 in the femurs of neonatal P1, P7, P14 and P21 (day 1, 7, 14 and 21) and adult 8‐weeks male C57BL/6 mice. The boxed representative images are under low magnification, and the larger images are the representative photographs under higher magnification (n=3/group). (B) The H&E and IHC staining of neonatal P7 (day 7) C57BL/6 mice tibia. Boxed area in H&E image represented the region that IHC staining was shown in high magnification. (C) IHC staining showing the expression of GPX7 in the femurs harvested at two weeks post‐fracture and its non‐fracture counterpart. The boxed representative images are under low magnification, and the larger images are the representative photographs under higher magnification (n=3/group). (D) The H&E and IHC staining of C57BL/6 mouse femurs harvested at two weeks post‐fracture. Boxed area in H&E image represented the region that IHC staining was shown in high magnification. (E) The protein levels of GPX7 in hBMSC and M2‐10B4 cells with osteogenic induction for 0, 1, 4 and 7 days. (F) The gene expression levels of *Gpx* families during osteogenesis in hBMSC and M2‐10B4 cells. Significance was determined via the Student's t test; data were represented as mean ±SD. *p < 0.05

To investigate whether GPX7 was expressed during bone development, we examined its expression in neonatal mice (day 1, 7, 14 and 21) and adult mice (8 weeks) femurs using IHC. The result showed that GPX7 was highly expressed in the cells located at the ossification centre below the growth plate in the femur metaphysis (Figure 1A). And the expression was down‐regulated with the completion of bone development. The expression level at 8 weeks was significantly lower than that at neonatal period. Besides, GPX7^+^ cells were coincident cells that were positive for Osterix (OSX), a transcription factor that is essential for osteoblast differentiation, in adjacent tissues of neonatal mouse tibiae (Figure 1B), suggesting the important role of GPX7 in osteogenesis during bone development.

In addition, to detect the expression of GPX7 during bone fracture repair, we constructed a mouse model of femur fracture. The IHC results showed that the expression of GPX7 in the bony‐callus area of femur harvested at two weeks post‐fracture is significantly higher than that in its non‐fracture control counterpart (Ctrl; Figure 1C). Consistently, GPX7 was distinctly expressed in OSX^+^ osteoblast‐like cells in the bony‐callus area (Figure 1D), indicating a high level of GPX7 activity during osteogenesis.

Given these findings, we performed experiments to elucidate the potential role of GPX7 in BMSCs mineralization. hBMSCs and M2‐10B4, a BMSC line, were subjected to differentiation conditions and harvested at different time points. We performed Western blotting analysis to identify the increased GPX7 levels in hBMSCs and M2‐10B4 cells (Figure 1E) during osteoblastogenesis. Besides, we have examined the transcriptional expression of GPX family during osteogenesis in hBMSCs and M2‐10B4 cells (Figure 1F). Interestingly, *Gpx3* was also found increased in hBMSCs during osteogenesis induction. But using siRNA‐*Gpx3* did not affect the osteogenic differentiation of hBMSC in vitro (data are not shown), and in M2‐10B4 cells, *Gpx3* had low expression. In summary, the above results suggested a crucial role of GPX7 during osteogenesis.

### 3.2 GPX7 is indispensable for osteogenic differentiation

**FIGURE 2:**
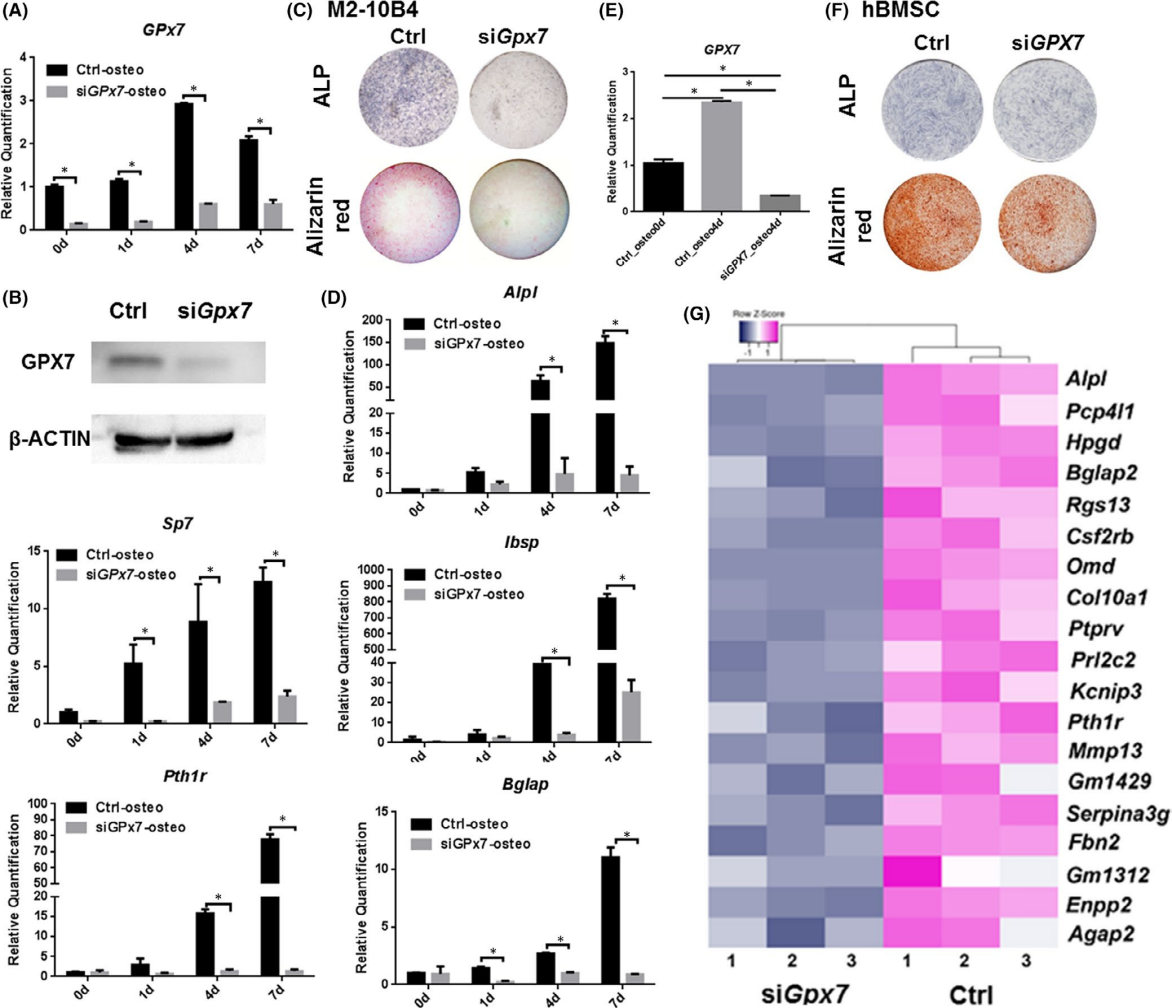
GPX7 is indispensable for osteogenic differentiation. (A‐B) Knockdown of *Gpx7* by siRNA in M2‐10B4 cells was verified at the gene and protein expression level. (C) The ALP staining (osteogenic induction for 4 days) and alizarin red staining (osteogenic induction for 14 days) shown that si*Gpx7* decreased the osteoblast differentiation potential of M2‐10B4 cells. (D) Osteogenic marker expressions were declined by *Gpx7* silencing in M2‐10B4 cells. (E) The expression of *Gpx7* after osteogenic induction and knockdown in hBMSCs. (F) The ALP staining (osteogenic induction for 4 days) and alizarin red staining (osteogenic induction for 7 days) shown that si*Gpx7* decreased the osteoblast differentiation potential of hBMSCs.G, RNA profiling of M2‐10B4 cells treated with si*Gpx7* over scramble was performed by RNA‐sequencing 4 days after osteogenic induction. The top 20 down‐regulated genes were listed by the heat map. Significance was determined via the Student's t test and one‐way ANOVA; data were represented as mean ±SD. *p < 0.05

To determine whether increased GPX7 levels play a functional role in osteoblast differentiation, we silenced *Gpx7* in both M2‐10B4 cells and hBMSCs via transduction of siRNA (Figure 2A&B; Figure S2A; Figure 2E). *Gpx7*‐silenced (si*Gpx7*) cells were subjected to either OM or growth medium, and we assessed osteoblastogenesis via ALP staining, alizarin red staining, as well as gene expression. As expected, impaired mineralization was observed in both M2‐10B4 cells and hBMSCs with *Gpx7* knockdown compared with the control counterparts (Figure 2C; Figure 2F). Besides, the silencing of *Gpx7* significantly decreased the expression level of *Alp*, *Sp7* transcription factor 7 (*Sp7*), integrin‐binding sialoprotein (*Ibsp*), bone gamma‐carboxyglutamate protein (*Bglap*), parathyroid hormone 1 receptor (*Pth1r*) (Figure 2D; Figure S1) and other osteogenic‐related genes, including GLI family zinc finger 1 (*Gli1*), patched 1 (*Ptch1*), insulin‐like growth factor 1 (*Igf1*), insulin‐like growth factor 2 (*Igf2*) and fibroblast growth factor 2 (*Fgf2*), as indicated by RT‐qPCR (Figure S2B).

Next, to investigate changes in RNA profiling in response to *Gpx7* silencing, we performed RNA sequencing of cells pretreated to si*Gpx7* and then incubated the cells in the OM for 4 additional days (Figure 2G). Of the top 20 significantly down‐regulated genes, *Alpl* was the most decreased, while other osteogenic markers such as *Bglap*, osteomodulin (*Omd*), Collagen Type X Alpha 1 Chain (*Col10a1*), *Pth1r* and matrix metallopeptidase 13 (*Mmp13*) were also dramatically reduced. In addition, enhanced adipogenic capacity was detected in both M2‐10B4 cells and hBMSCs with *Gpx7* knockdown when compared with the control groups (Figure S3), and adipogenesis is often inversely related to osteogenesis when it comes to cell fate determination.^32^ These findings demonstrated that *Gpx7* was indispensable to BMSC osteogenesis and calcification.

### 3.3 Silence of *Gpx7* has no effects on cell proliferation, cell cycle or apoptosis

**FIGURE 3:**
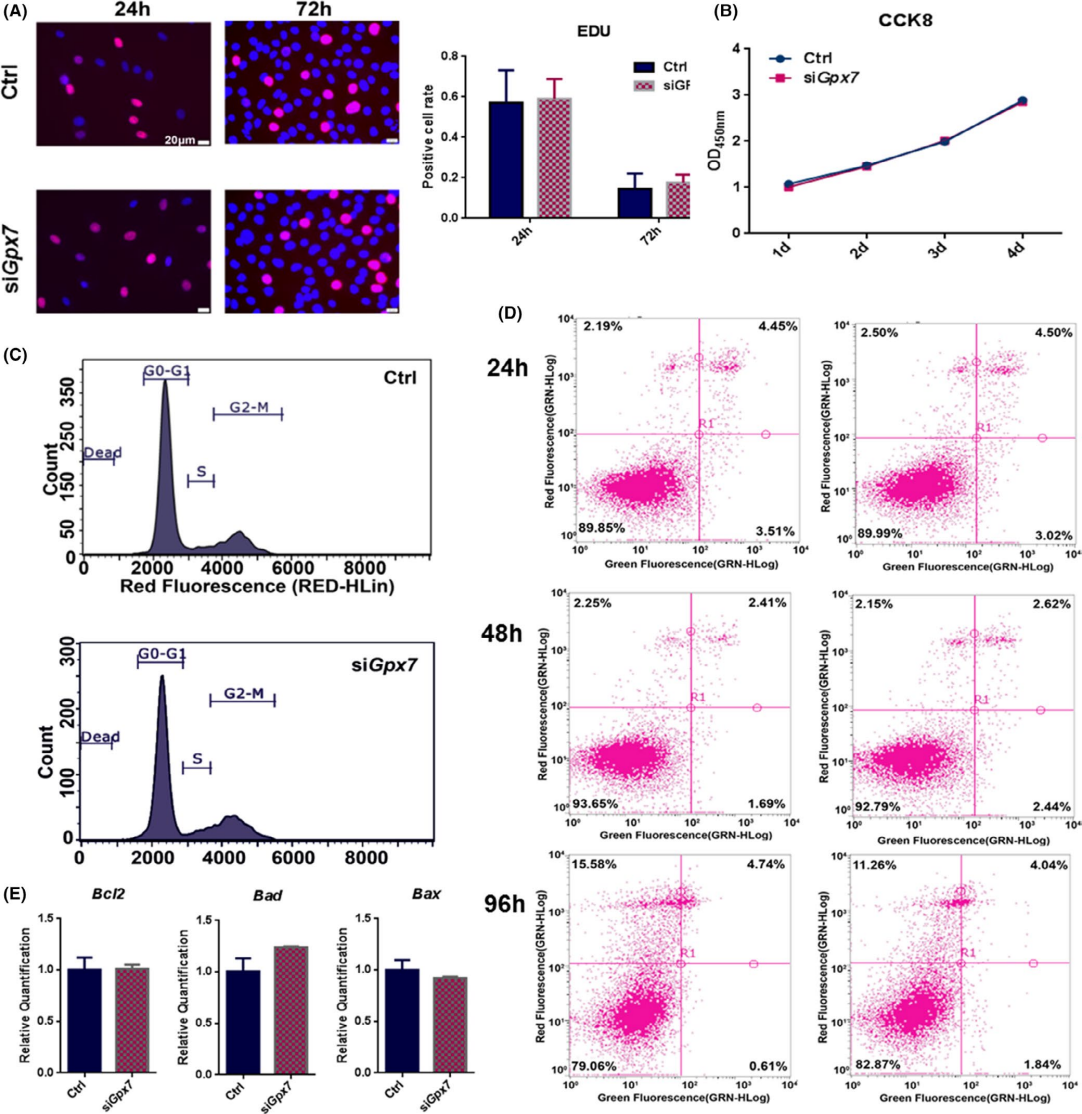
Knockdown of *Gpx7* does not change cell proliferation, cell cycle and apoptosis. (A&B) The cell proliferation of si*Gpx7* and Ctrl cells was measured by CCK8 and EdU staining; (C) PI staining and flow cytometry were used to detect the cell cycle in both conditions after 24 hrs of *Gpx7* knockdown. (D) Apoptosis was detected by Annexin V‐FITC/PI staining and flow cytometry analysis after 24 hrs, 48 hrs and 96 hrs of *Gpx7* knockdown. (E) The expressions of apoptotic genes, *Bcl2*, *Bad* and *Bax*, were detected 24 hrs later after the knockdown of *Gpx7*. Significance was determined via the Student's t test; data were represented as mean ±SD. *p < 0.05

GPX7 is an important member of the GPXs family. Previous studies have suggested that it plays an important role in the anti‐stress ability of cells.^33^ Therefore, we investigated whether *Gpx7* down‐regulation disrupted physiological functions such as proliferation, apoptosis and cell cycle under basic culture conditions in M2‐10B4 cells. Immunostaining with 5‐ethynyl‐2'‐deoxyuridine (EdU) and cell counting kit 8 assay results showed that si*Gpx7* did not affect cell proliferation under basal conditions (Figure 3A, B). Similarly, as per flow cytometry analysis results after Annexin V‐fluorescein isothiocyanate (FITC)/propidium iodide (PI) staining, we saw no significant differences in cell cycle or apoptosis under the indicated regiments (Figure 3C, D) between the two groups. However, we observed no dramatic differences in B‐cell lymphoma 2 (*Bcl2*), *Bcl*‐*2*‐associated agonist of cell death (*Bad*) or *Bcl*‐*2*‐like protein 4 (*Bax*) expression after *Gpx7* knockdown for 24 hrs (Figure 3E). These results indicated that *Gpx7* deficiency did not change cell proliferation, apoptosis and cell cycle under basal culture conditions.

### 3.4 Inhibition of ROS did not rescue the decline of osteogenesis caused by *Gpx7* deficiency

**FIGURE 4:**
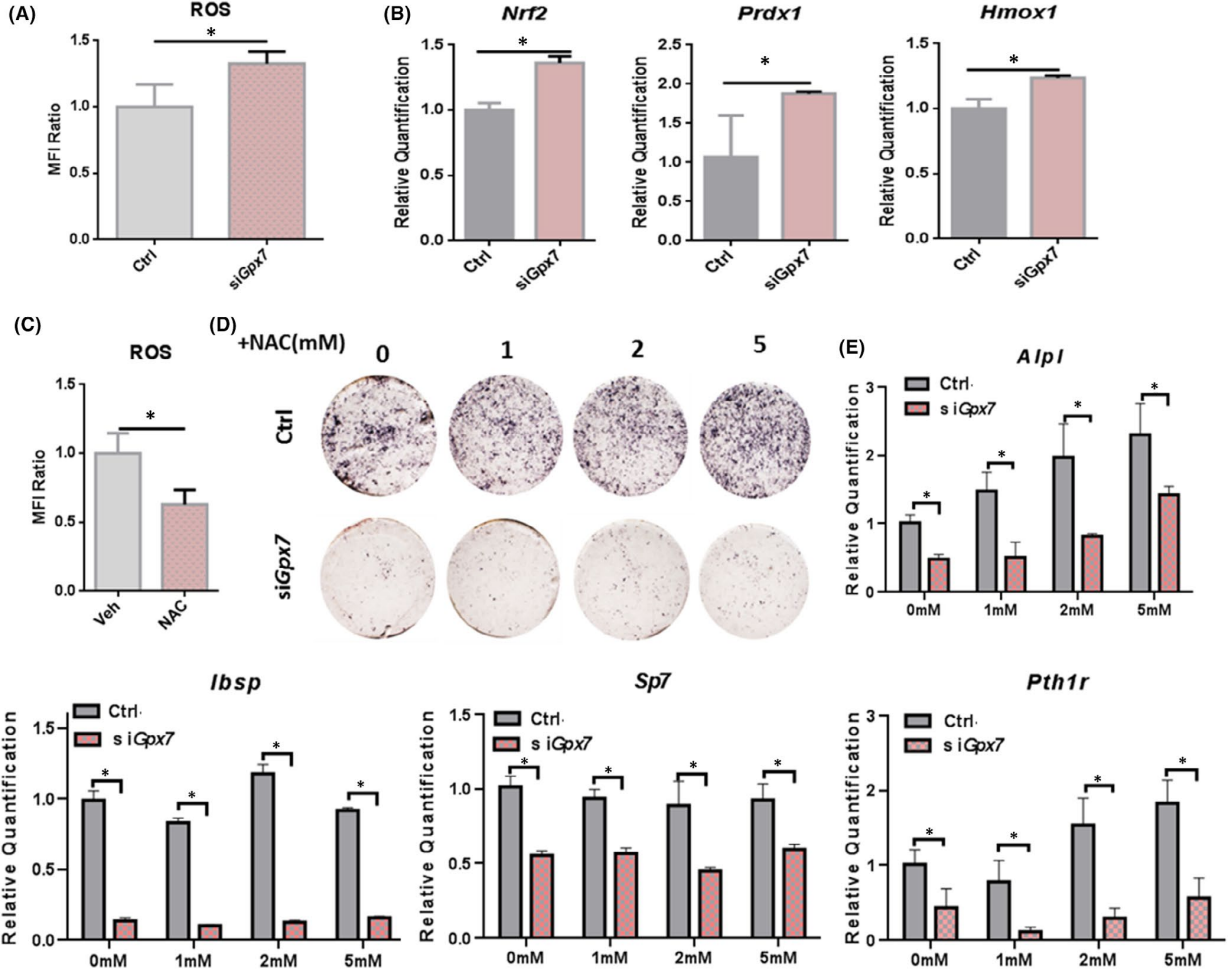
*Gpx7* knockdown leads to the up‐regulation of ROS, but NAC cannot restore the decline of osteogenesis. (A) The intracellular ROS contents in the si*Gpx7* and Ctrl cells were detected after knockdown of *Gpx7*. (B) The expression of OS‐related gene *Nrf2*, *Prdx1* and *Hmox1* was measured by RT‐qPCR. (C) The intracellular ROS contents were tested after cells exposing to vehicle (Veh) or 5mM antioxidant NAC for 24 hrs. (D&E) Cells were pre‐treated with vehicle or NAC for 24 hrs, followed by osteogenic induction for 4 additional days. Then, the ALP staining (D) and the expression level of osteoblast marker genes (E) were performed. Significance was determined via the Student's t test; data were represented as mean ±SD. *p < 0.05

Previous studies have shown that GPX7 deficiency led to the up‐regulation of intracellular ROS and OS,^9^ which was thought to be responsible for obesity in mice and humans with GPX7 deficiency.^13^ Therefore, in this study, we investigated whether the up‐regulation of ROS was also associated with decreased osteogenesis in M2‐10B4 cells. Consistent with previous studies, we found that intracellular ROS was up‐regulated 6 hrs after *Gpx7* knockdown (Figure 4A). Subsequently, we examined the OS genes expression and found that nuclear factor (erythroid‐derived 2)‐like 2 (*Nrf2*), peroxiredoxin 1 (*Prdx1*) and haeme oxygenase 1(*Hmox1*) were up‐regulated 24 hrs after *Gpx7* knockdown (Figure 4B). Meanwhile, the protein level of NRF2 was also increased 48 hrs after *Gpx7* knockdown (Figure S4). This suggested that the intracellular antioxidant‐stress process had been initiated after *Gpx7* knockdown. These data were consistent with our previous finding that intracellular ROS content was relatively up‐regulated in the si*Gpx7* group after an early elevation at 6 hrs.

Therefore, to explore whether this reduction in osteogenic potential was due to the early increase in ROS, we found that adding 5 mM antioxidant NAC significantly inhibited intracellular ROS (Figure 4C). Then, we added NAC to the cells which previously seeing si*Gpx7* for 24 hrs prior to osteogenic induction for 4 additional days. Osteogenic differentiation was assessed by ALP staining (Figure 4D) and RT‐qPCR (Figure 4E) assays. Interestingly, the results showed that the addition of NAC did not restore the osteogenic defect due to *Gpx7* silencing, suggesting that increasing ROS was not the primary cause for the decline of osteogenesis.

### 3.5 *Gpx7*‐knockdown‐induced ER stress leads to osteogenic differentiation defect

**FIGURE 5:**
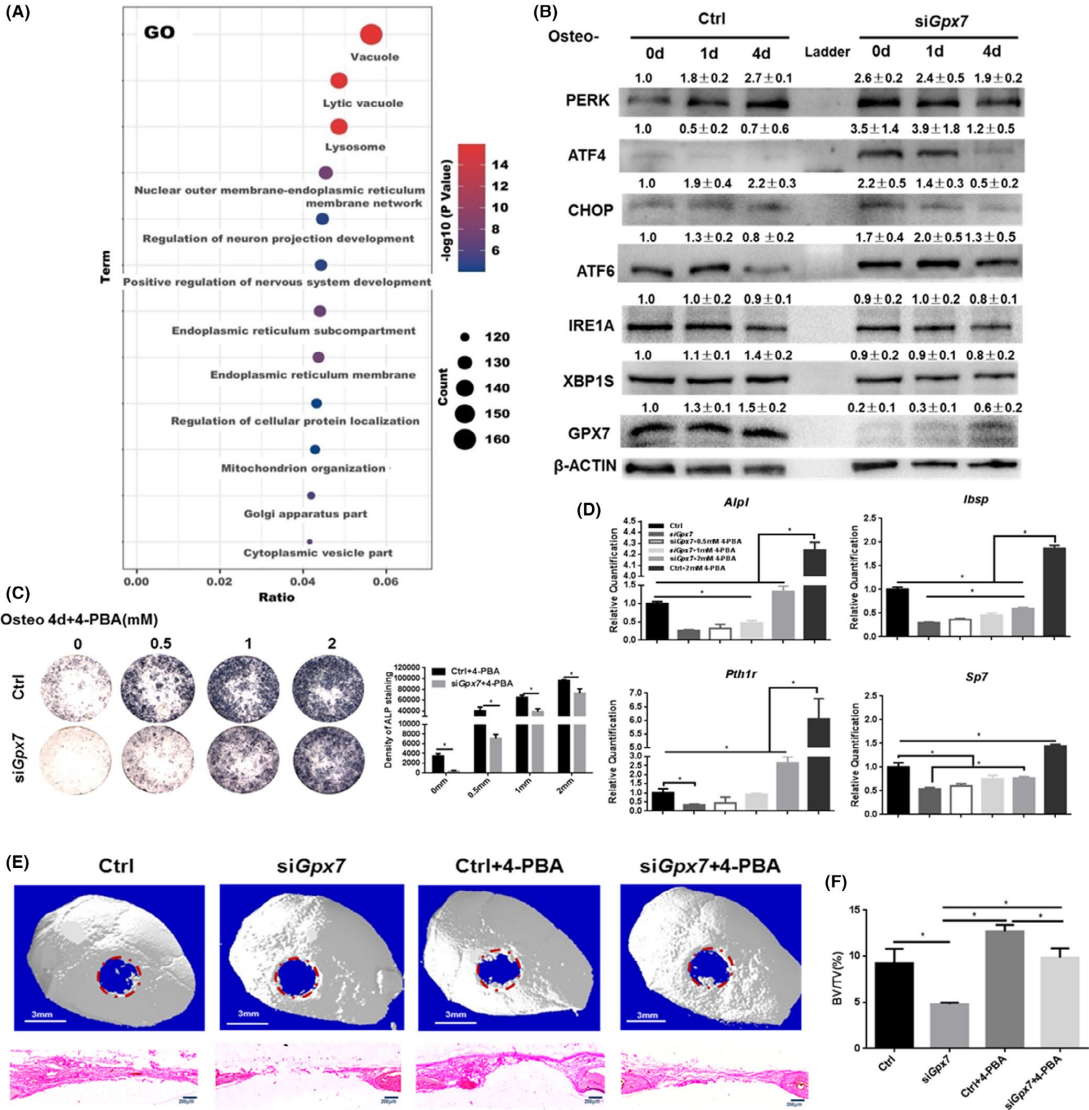
Accumulation of ER stress caused by G*px7* deficiency hampers osteoblastogenesis. (A) The GO analysis of the RNA sequencing with M2‐10B4 cells under either si*Gpx7* or scramble condition, followed by osteogenesis for another 24 hrs. (B) Western blotting of ER stress‐related proteins. The integrated density of blots was measured by ImageJ and normalized to β‐ACTIN. The fold changes were calculated from three independent experiments with SD indicated. Osteo‐OM, osteogenic medium. C&D The ALP staining (C) and osteogenic marker gene expressions (D) were detected in si*Gpx7* cells after 4‐PBA administration for 24 hrs followed by osteoblast induction for 4 additional days. (E) Representative micro‐CT 3D‐reconstruction images of calvarial bone defects at 4 weeks post‐implantation and the specimens stained with H&E. (F) The BV/TV analysis of the newly formed bone. Significance was determined via the one‐way ANOVA; data were represented as mean ±SD. *p < 0.05

To investigate the mechanisms by which *Gpx7* deficiency resulted in the deterioration of BMSC mineralization, we performed RNA sequencing by using M2‐10B4 cells pretreated with the si*Gpx7* or scramble and then incubated in the OM for additional 24 hrs. Gene Ontology (GO) analysis showed that the ER‐related gene function groups were the most enriched of the top 12 groups after *Gpx7* knockdown (Figure 5A). GPX7 is mainly located in the ER, and excessive ER stress is thought to inhibit osteogenesis.^34^ In addition, previous studies have shown that GPX7 is associated with the UPR process,^35^, ^36^ which is a major component of ER stress. Therefore, it is important to demonstrate whether the down‐regulation of osteogenic differentiation caused by *Gpx7* knockdown was due to the increased ER stress.

To this end, we first detected the expression of ER stress‐related proteins with *Gpx7* knockdown. Western blotting results showed that UPR‐related proteins, such as PERK, ATF4, CHOP and ATF6, were indeed significantly up‐regulated after 24 hrs of *Gpx7* knockdown, but IRE1a and XBP1s were not significantly changed. During osteogenesis, ATF4 and ATF6 remained increasing in the absence of *Gpx7*, but PERK and CHOP were decreased which may be due to the ER stress compensation (Figure 5B). Subsequently, we added 4‐PBA, an ER stress inhibitor that enhances protein folding ability, to both si*Gpx7* and scramble cells for 24 hrs prior to osteogenic induction. We found that it partially restored osteogenic defects in the si*Gpx7* cells, indicating ALP staining and gene expression detection (Figure 5C&D). Furthermore, dose‐dependently introducing 4‐PBA enhanced osteogenic gene expression of *Alpl*, *Sp7* and *Pth1r in vitro* (Figure S5A), while adding ER stress inducer tunicamycin (TM) dose‐dependently repressed such expressions (Figure S5B). These results suggested that the knockdown of *Gpx7* impeded osteogenesis by enhancing ER stress *in vitro*.

To further confirm the hypothesis *in vivo*, we performed calvarial bone defects in male C57BL/6 mice. In this assay, mBMSCs were infected with Ad‐*Gfp* or Ad‐si*Gpx7*, followed by administration with or without 2 mM 4‐PBA (Ctrl, Ctrl+4‐PBA, si*Gpx7* and si*Gpx7*+4‐PBA, respectively). Those cells were then mixed with Germa gel, which were next transplanted to the bone defect area. The micro‐CT 3D‐reconstruction, H&E staining and morphometric analysis BV/TV results showed that the area of newly formed bone tissue in the si*Gpx7* group was significantly lower than that of Ctrl, Ctrl+4‐PBA and si*Gpx7*+4‐PBA groups, in which almost no newly formed bone was observed. While in other three groups, the newly formed bone tissue can be observed in the lateral margin and the newly formed tissue in the si*Gpx7*+4‐PBA group was similar to that of the Ctrl group (Figure 5E&F), which demonstrated that the new bone formation in the defect area was impaired by GPX7 deficiency, and this defect can be rescued by relieving ER stress with 4‐PBA. In summary, these data suggested that *Gpx7* silencing enhanced ER stress, which in turn elicited osteogenic defects in BMSCs *in vitro* and *vivo*.

### 3.6 *Gpx7* promoted osteogenesis by maintaining the function of the mTOR pathway

The mTOR signalling is not only critical for osteoblastogenesis, but also essential for sensing ER stress.^25^, ^37^, ^38^ In addition to inducing ER stress, we found that *Gpx7* knockdown also repressed the intracellular mTOR pathway, which is essential for osteogenesis.^30^, ^39^

**FIGURE 6:**
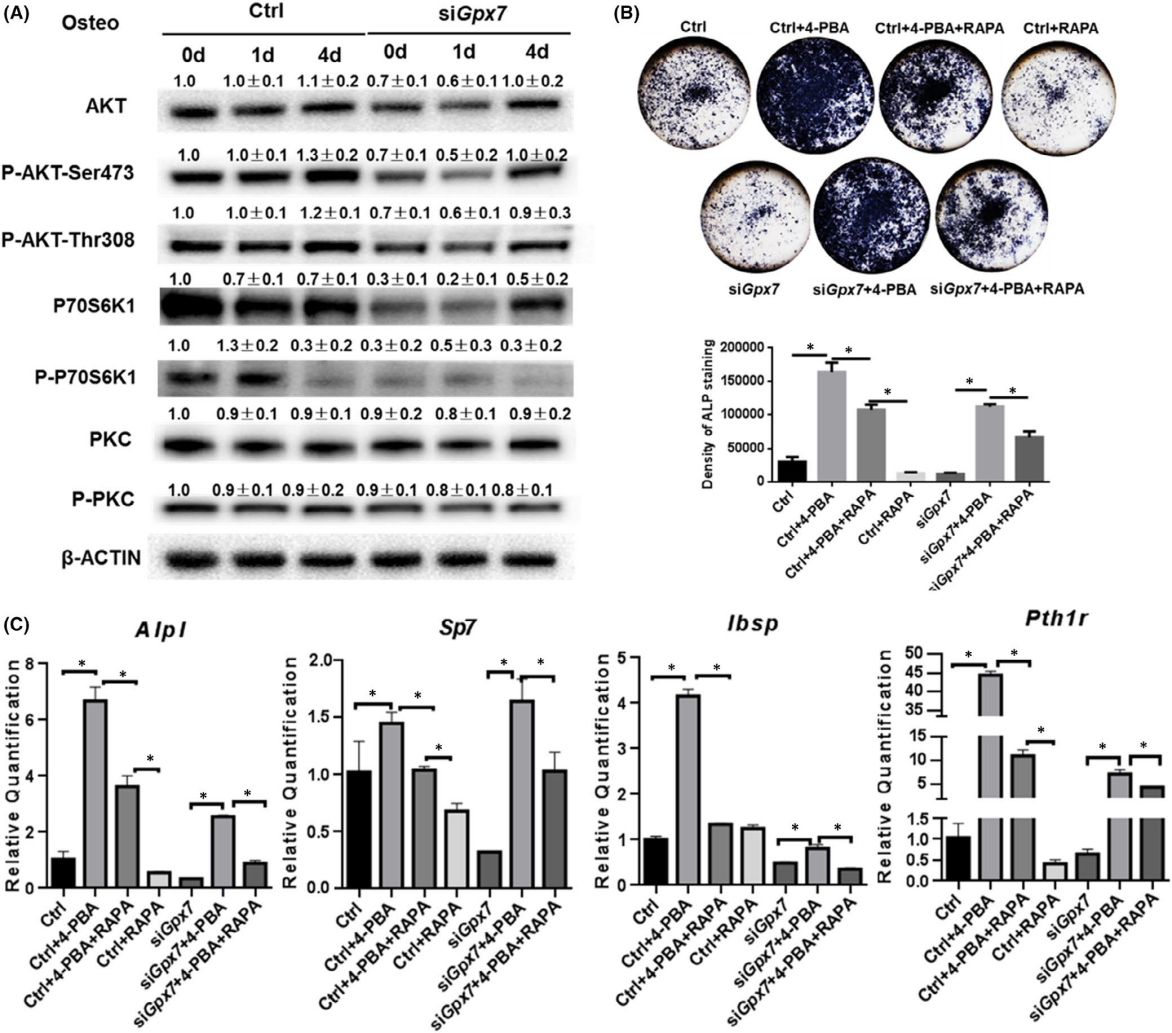
GPX7 deficiency caused osteogenic defect is mediated by mTOR and ER stress. (A) Western blotting of the proteins which were involved in the mTOR pathway. The integrated density of blots was measured by ImageJ and normalized to β‐ACTIN. The fold changes were calculated from three independent experiments with SD indicated. (B&C) The ALP staining (B) and the expression level of osteoblast marker genes (C) in M2‐10B4 cells after RAPA were added for 6 hrs with the following osteoblast induction for 4 days, with or without 2 mM 4‐PBA treatment in Ctrl and si*Gpx7* cells. Significance was determined via the one‐way ANOVA; data were represented as mean ±SD. *p < 0.05

M2‐10B4 cells were treated with *Gpx7* and scramble siRNA for 24 hrs and then exposed to osteogenic medium for 1 or 4 additional days. Western blotting experiments showed that due to the *Gpx7* deficiency, the protein levels of AKT, phosphorylated AKT (P‐AKT), P70S6K1 and phosphorylated P70S6K1 (P‐P70S6K1) were strikingly suppressed. We also saw a slight decrease in phosphate kinase C (PKC) and phosphorylated PKC (P‐PKC) 24 hrs after *Gpx7* knockdown, followed by osteogenic induction for 1 additional day. These results indicated that GPX7 was indispensable for mTOR signalling (Figure 6A). Previous studies have shown that enhanced ER stress initiates a decline in mTOR pathway function.^26^ Therefore, we speculated whether *Gpx7* knockdown would also cause such a decline due to increased ER stress. To demonstrate this, after *Gpx7* silencing, we added 1 μg/mL rapamycin (RAPA), an inhibitor of the mTOR pathway, to the cells with or without 2 mM 4‐PBA for 6 hrs. Cells then underwent osteogenesis for 4 days. RAPA was found to impair osteogenesis in both the si*Gpx7* and the control groups as expected (Figure 6B, C). The ALP staining and RT‐qPCR results showed that consistent with our previous finding, *Gpx7* deficiency caused deterioration of osteogenesis, which was partially restored by eliminating ER stress using 4‐PBA. Moreover, this rescue was significantly abolished when the mTOR complex was interrupted (Figure 6B, C). Taken together, these findings indicated that *Gpx7* facilitated osteoblastogenesis by eliminating ER stress**‐**related misfolding, which sequentially enabled the mTOR pathway to remain intact and to function.

## 4 DISCUSSION

Obesity has been identified as a global epidemic caused by over‐nutrition or genetic defects. Obese individuals exhibit not only overweight and energy imbalance but also a dramatic increase of fat accumulation in the body and a decrease in bone mass.^40^ Besides, obesity is associated with the accumulation of ROS and misfolded proteins generated due to ER stress, which decreases the whole body's energy level^41^ and also leads to aberrant lineage allocation of mesenchymal stem/progenitor cells (MSPC).^42^, ^43^

Numerous studies have confirmed the promising role of BMSCs, a type of MSPC, in stimulating bone regeneration and remedying bone defects. BMSCs can be induced to differentiate into a variety of cell types, including osteoblasts and adipocytes.^44^ Increasing adipocytic differentiation decreases osteoblastic differentiation and *vice versa*.^32^ Despite extensive studies, the mechanism governing BMSC fat determination is still not fully understood.

GPX is one of the most important enzyme families involved in the antioxidant‐stress process. Of note, *Gpx7* deficiency is believed to increase intracellular OS and especially ROS levels, which elevates adipogenesis together with white adipose‐tissue mass, thereby leading to obesity in both mice and humans.^13^ In addition, we screened *Gpx7*’s published online gene profile and, interestingly, found that its expression was significantly increased during osteogenesis.^14^ Taken together, these data indicated that *Gpx7* might play a role in BMSC differentiation.

To confirm this hypothesis and to verify whether *Gpx7* was involved in the determination of BMSC fate, we analysed the expression of *Gpx7* during differentiation of BMSCs and performed functional experiments. This study provides the first evidence that *Gpx7* facilitated osteogenesis but inhibited adipogenesis in BMSCs. From a mechanistic standpoint, we wondered whether the inhibition of osteogenesis caused by *Gpx7* deficiency was due to defects in ROS homeostasis. However, to our surprise, although the knockdown of *Gpx7* led to the up‐regulation of ROS level and higher expression of OS genes in the early phase, administration of the antioxidant NAC did not restore the osteogenic defects. Based on this finding, we speculated that, unlike adipogenesis, ROS up‐regulation is not the main factor leading to the decline in osteogenesis; other factors might also restrict osteogenic differentiation.

Therefore, to identify the molecular mechanism by which *Gpx7* deficiency suppressed osteogenesis, we conducted mRNA sequencing in the early stage of osteogenic induction after *Gpx7* knockdown. Analysis of our data showed that the expression of ER‐associated genes was most significantly reduced in response to *Gpx7* deficiency. Previous studies have shown that GPX7 can bind to glucose‐regulated protein 78 (GRP78) and protein disulphide isomerase (PDI), other ER‐resident proteins that serve as chaperones to promote refolding of faulty proteins and maintain redox homeostasis in the ER.^9^, ^35^, ^36^ Accumulation of misfolded proteins can lead to ER stress, which can cause translation attenuation, up‐regulation of chaperones and degradation of misfolded proteins.^45^ The relationship between ER stress and osteogenesis has been widely reported; excessive ER stress is thought to inhibit osteogenesis.^34^ During osteogenic differentiation, it is necessary to produce a large number of extracellular‐matrix (ECM) proteins; the secreted proteins are synthesized and acquire their three‐dimensional conformation in the ER. Therefore, functional ER is essential for osteogenesis.^46^ Other previous studies have shown that *Gpx7* deficiency can attenuate the efficiency of chaperone activity against misfolded proteins and exacerbate misfolded‐protein accumulation in ER.^9^ In our study, we observed increased ER stress in *Gpx7*‐deficient M2‐10B4 cells by detecting UPR sensor proteins, mainly PERK, ATF4 and ATF6, which are indicators of ER stress. Moreover, the exogenous addition of ER stress inhibitor 4‐PBA could partly rescue the decline in osteogenesis *in vitro* and *vivo*. Therefore, we speculated that *Gpx7* might be involved in osteogenesis by, at least in part, regulating ER stress.

During the determination of BMSC fate, the mTOR signalling pathway not only serves as a switch for differentiation but is also critical for sensing ER stress.^25^, ^37^, ^38^ In addition to ER stress, we also found the mTOR pathway to be down‐regulated when *Gpx7* was deficient. This pathway is a master regulator of cell growth, metabolism and survival. It contains two complexes, mTORC1 and mTORC2.^31^ Down‐regulation of mTOR signalling can lead to decreased osteogenic ability,^28^ as osteoblastic differentiation requires the participation of protein anabolism and related mRNA translation.^47^ Notably, the down‐regulation of mTOR pathway was mainly due to the decrease of total AKT and P70S6K1, rather than a change in the phosphorylation modification (P‐AKT and P‐P70S6K1) in our study, which suggested that *Gpx7* might be involved in the regulation of total AKT and P70S6K1 in cells. Interestingly, in our study, the decline in mTOR pathway function could be restored by the addition of 4‐PBA, which indicated that such decline might be caused by increased ER stress in the presence of *Gpx7* deficiency. This was consistent with the findings of previous studies that prolonged chronic ER stress can inhibit both mTORC1 and mTORC2.^25^ Taken together, these experimental results indicated that *Gpx7* deficiency might lead to a reduction in mTOR pathway function by inducing up‐regulation of ER stress, thereby reducing the osteogenic potential of BMSCs.

In summary, in this work, we have provided the first evidence of the role played by *Gpx7* during osteoblastic differentiation and identified this gene as regulating osteogenic differentiation by regulating ER stress and the mTOR pathway. These findings will establish the critical role of *Gpx7* in osteogenesis and serve to identify the potential mechanism underlying BMSC osteogenic differentiation.

## CONFLICT OF INTEREST

All authors state that they have no conflicts of interest.

## AUTHOR CONTRIBUTIONS

**Xuchen Hu:** Data curation (lead); Formal analysis (lead); Investigation (equal); Methodology (equal); Validation (lead); Writing‐original draft (equal). **Boer Li:** Conceptualization (supporting); Methodology (supporting); Project administration (supporting); Visualization (supporting); Writing‐review & editing (supporting). **Fanzi Wu:** Methodology (supporting); Project administration (supporting); Resources (equal); Software (supporting); Writing‐review & editing (supporting). **Xiaoyu Liu:** Methodology (equal); Project administration (supporting); Writing‐review & editing (supporting). **Mengyu Liu:** Methodology (equal); Project administration (supporting); Resources (supporting); Writing‐review & editing (supporting). **Chenglin Wang:** Conceptualization (supporting); Methodology (supporting); Supervision (equal); Validation (supporting); Writing‐review & editing (equal). **Yu Shi:** Conceptualization (lead); Funding acquisition (supporting); Supervision (equal); Writing‐original draft (equal); Writing‐review & editing (lead). **Ling Ye:** Conceptualization (lead); Funding acquisition (lead); Investigation (equal); Supervision (equal); Writing‐review & editing (lead).

## Supporting information

Supporting Information

## Data Availability

The data that support the findings of this study are available from the corresponding author upon reasonable request.
