# Supplementary material for: GPX7 Facilitates BMSCs Osteoblastogenesis via ER Stress and mTOR Pathway
Source: J Cell Mol Med. 2021 Oct 9;25(22):10454–65. doi: 10.1111/jcmm.16974 (PMC8581313; doi:10.1111/jcmm.16974)
Supplement: Supplementary file 1 — Supporting Information [file JCMM-25-10454-s001.docx]

**Supporting Information**

**GPX7 Facilitates BMSCs Osteoblastogenesis via ER Stress and mTOR Pathway**

Xuchen Hu^1^, Boer Li^1^, Fanzi Wu^1^, Xiaoyu Liu^1^, Mengyu Liu^1^ , Chenglin Wang^1^, Yu Shi^1,2^, Ling Ye^1,2^

**SI Methods**

**Small-interfering RNAs and cell transfection.**

The small-interfering RNA (siRNA) for GPX7 (si*Gpx7*-RNA) and negative control siRNA (Ctrl-RNA) were synthesized from Hanbio Inc. (Shanghai, China), and the sequences are as follows: m*Gpx7*: GAAACTCATCCTTCGGAAACG; and h*GPX7*: GCUUCACAGACCAGCACUATT. The siRNAs were transfected with Lipofectamine 3000 (L3000150, Thermo Scientific, MA, USA) according to the manufacturer’s protocol. Cells received Ctrl-RNA, si*Gpx7*-RNA for 12 hrs, then the medium was replaced by fresh complete medium or conditioned medium.

**Quantitative Real-time PCR analysis (RT-qPCR)**

Total RNAs were isolated from M2-10B4 cells and hBMSCs by Trizol extraction (Invitrogen, Carlsbad, CA, USA), complementary DNA was synthesized by using the

instructions. RT-qPCR was performed with SYBR Premix Ex Taq (Takara, Dalian, China) on a Real-Time PCR System with analytical software (BioRad, USA). Primers used for RT-qPCR are listed in Table S1.

**Western blotting assay**

For Western blotting, cells were lysed with M-PER™ Mammalian Protein Extraction Reagent (Thermo Scientific, IL, USA) containing Halt™ Protease and Phosphatase Inhibitor Cocktail (Thermo Scientific, IL, USA) on ice for 5 minutes. Cell lysates were centrifuged at 13,000×g for 20 min at 4 °C. Equivalent proteins as determined by BCA assay (Beyotime, Shanghai, China) were resolved by SDS-PAGE and subjected to Western blotting with appropriate primary antibodies (1:500-2000), including PERK (Proteintech, 20582-1-AP), ATF4 (Proteintech, 10835-1-AP), ATF6 (ZENBIO, 500202), IRE1A (Cell Signaling Technology Inc, 3294S), XBP1s (Cell Signaling Technology Inc, 40435S), CHOP (Proteintech, 15204-1-AP), GPX7 (Proteintech, 13501-1-AP), β-ACTIN (Proteintech, HRP-60008), AKT (Cell Signaling Technology Inc, 9272S), P-AKT-Thr308 (Cell Signaling Technology Inc, 13038S), P-AKT-Ser473 (ZENBIO, 310021), P70S6K1 (Proteintech, 14485-1-AP), P-P70S6K1 (ZENBIO, R22939), PKC (ZENBIO, 385382), P-PKC (ZENBIO, R22939). Signals from the blots were visualized with enhanced chemiluminescence (Thermo Scientific, IL, USA) with the Bio-Rad detection system (Bio-Rad, Hercules, CA).

**High-throughput mRNA sequencing**

The total RNA samples from Ctrl and si*Gpx7* cells (osteogenic induction for 1 or 4 days), were extracted separately using TRIzol and the RNeasy mini kit (Qiagen, Germany). After quality checks, the RNA samples were subjected to high-throughput RNA-sequencing (Novogene, Beijing, China). The data was analyzed by Novogene Inc. (Beijing, China). Each group consisted of 3 replicates. Genes with a fold change >1.5 and a *p* < 0.05 were considered to be differentially expressed. The accession number for the RNA-seq data reported in this paper is PRJNA724696.

**Cell proliferation, apoptosis and cell cycle assay**

Cell proliferation was evaluated by the Cell Counting Kit-8 (CCK-8; Dojindo, Kumamoto, Japan) and Cell-Light EdU Apollo 567 (RiboBio, Guangzhou, China) according to the manufacturer’s protocols. Briefly, M2-10B4 cells were seeded in 96-well plates and the Ctrl-siRNA or *Gpx7*-siRNA were added overnight to knock down the gene. Subsequently, CCK8 results were assessed by the addition of 10% CCK-8 and incubation for 2 h. The absorbance was measured at 450 nm. For EdU staining, the Ctrl-siRNA or *Gpx7*-siRNA cells were cultured in 96-well plates containing 10 μM EdU for 2 h at 37 °C for labeling. Hoechest 33342 was used for Nuclear staining. Images were captured with a Nikon Eclipse 300 fluorescence microscopy (CompixInc, Sewickley, PA).

The apoptosis rate was evaluated using the Annexin V-FITC/PI Apoptosis Detection kit (Biotime, Shanghai, China) according to the instructions from the manufacturer. The cells were seeded into 6-well tissue culture plates. Following treatment, the cells were collected and 5 μL Annexin V-FITC and 5 μL PI were added to the buffer and incubated at room temperature for 15 min in the dark. Cells were analyzed by flow cytometry (BD FACSCanto) within 1 h.

For the cell cycle, the cells were collected and washed with PBS. Cells were treated with Cell cycle analysis kit reagents (KeyGEN; Nanjing, China) and were detected by flow cytometry (BD FACSCanto). The percentage of cells in the G1 phase, the S phase, and the G2 phase was analyzed.

**Determination of ROS**

The intracellular levels of ROS were estimated with the fluorescent dye 2′7′-dichlorodihy-drofluorescein diacetate (H2DCFDA; Thermofisher, Waltham, MA). For measurement, the cells were loaded with 10 µM H2DCFDA according to the manufacturer’s instructions. After cells were incubated at 37 °C for 30 min in the dark, the dye was removed, and cells were washed once with PBS and all of the samples were measured using a Flow cytometer immediately


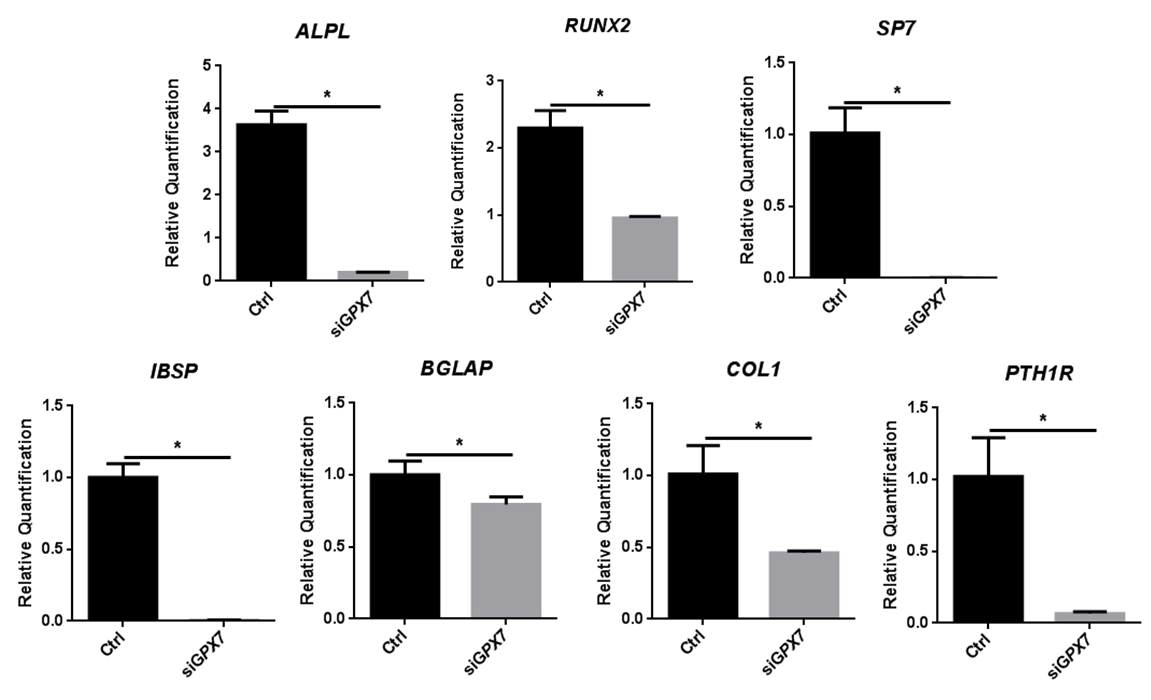


**FIGURE S1, GPX7 deficiency attenuates osteoblast differentiation in hBMSCs.** Silencing of *Gpx7* significantly decreases the expression of *Alpl*, *Runx2*, *Sp7*, *Ibsp*, *Bglap*, *Col1,* and *Pth1r* after osteogenic induction for 4 days in hBMSCs in vitro. Significance is determined via the student T-test; data are represented as mean ± SD. **P* < 0.05.


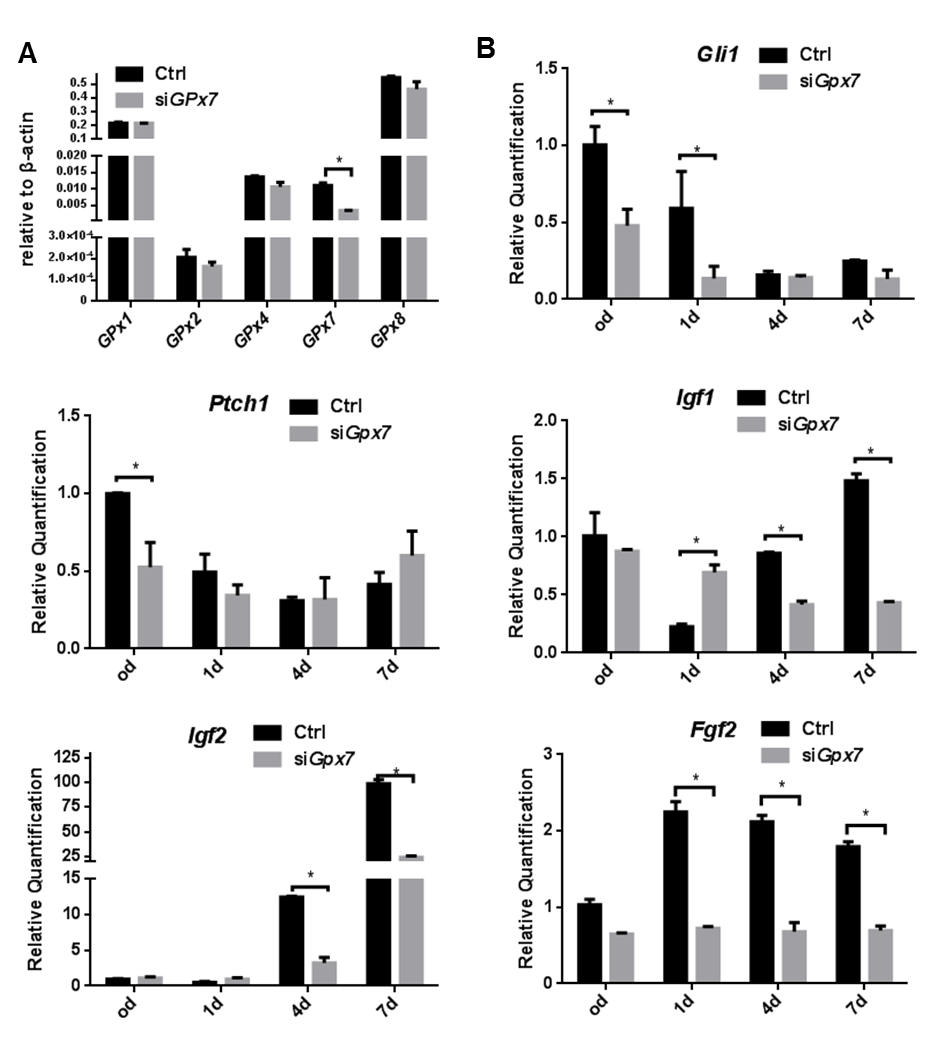


**FIGURE S2, Silencing of *Gpx7* significantly decreases the expression level of osteogenic related genes.**

(A) The gene expression levels of *Gpx* families are detected after *Gpx7* is silenced.

(B) The RT-qPCR results show that *Gpx7* deficiency significantly decreases the mRNA levels of *Gli1*, *Ptch1*, *Igf1*, *Igf2,* and *Fgf2* after osteogenic induction for 4 days in M2-10B4 cells.

Significance is determined via the student T-test; data are represented as mean ± SD. **p* < 0.05.


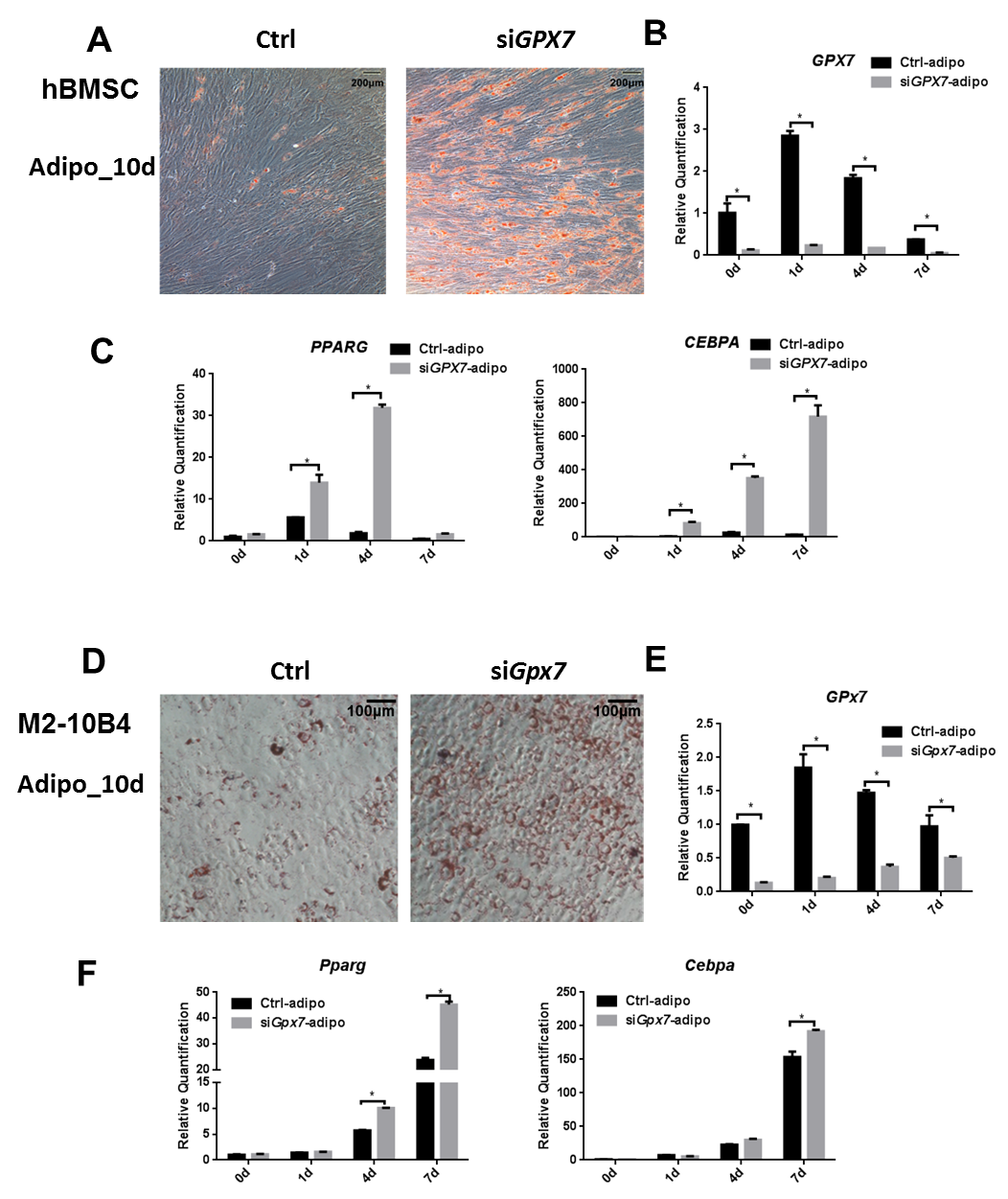


**FIGURE S3, GPX7 deficiency promotes adipogenesis.**

(A-C) The Oil red O staining (adipogenesis induction for 10 days) and RT-qPCR results show that *Gpx7* knockdown increases the adipogenesis potential of hBMSCs.

(D-F) The Oil red O staining (adipogenesis induction for 10d) and RT-qPCR results show that *Gpx7* knockdown increases the adipogenesis potential of M2-10B4 cells. Significance is determined via the student T-test; data are represented as mean ± SD. **P* < 0.05.


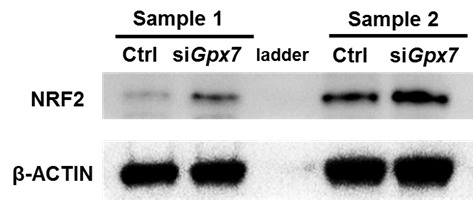


**FIGURE S4, *Gpx7* knockdown up-regulated NRF2**

The protein level of NRF2 in Ctrl and si*Gpx7* cells was shown by Western blotting.


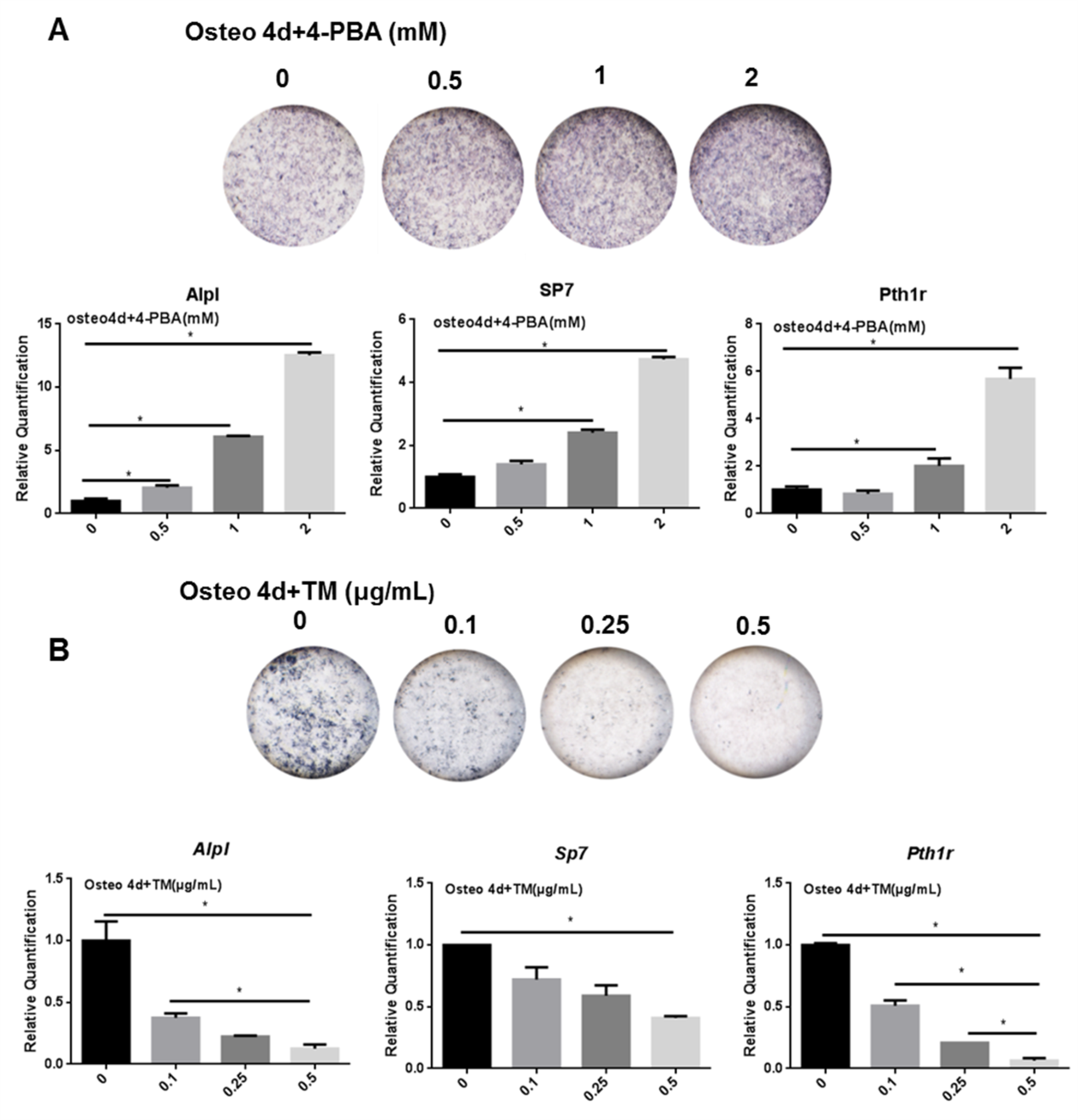


**FIGURE S5, ER stress inhibitor 4-PBA promotes osteogenic differentiation, while ER stress agonist TM inhibits mineralization.**

(A) The ALP staining and expression level of osteoblast marker genes in M2-10B4 cells after 4-PBA treatment for 24hrs prior to osteoblast induction for another 4 days. (B) The ALP staining and expression level of osteoblast marker genes in M2-10B4 cells after TM treatment for 24hrs prior to osteoblast induction for 4 additional days. Significance is determined via the One-way ANOVA; data are represented as mean ± SD. **P* < 0.05.

**Table S1, Primer sequences for qRT-PCR**

| **Gene** |  |  |
| --- | --- | --- |
| ***Mouse*** | **F** | **R** |
| ***Actb*** | CTCAGGAGGAGCAATGATCTTGAT | TACCACCATGTACCCAGGCA |
| ***18s*** | GCACTTTAGATGGACGAATCGC | TGCTGAGGCTAGATGAAACCA |
| ***Alpl*** | CCAACTCTTTTGTGCCAGAGA | GGCTACATTGGTGTTGAGCTTTT |
| ***Sp7*** | GGAAAGGAGGCACAAAGAAGC | CCCCTTAGGCACTAGGAG |
| ***Ibsp*** | ATGGAGACGGCGATAGTTCC | CTAGCTGTTACACCCGAGAGT |
| ***Pth1r*** | CAGGCGCAATGTGACAAGC | TTTCCCGGTGCCTTCTCTTTC |
| ***Bglap*** | TGCTGGAGTGGTCTCTATGA | ACCCTCTTCCCACACTGT |
| ***Gpx1*** | AGTCCACCGTGTATGCCTTCT | GAGACGCGACATTCTCAATGA |
| ***Gpx2*** | GCCTCAAGTATGTCCGACCTG | GGAGAACGGGTCATCATAAGGG |
| ***Gpx4*** | GATGGAGCCCATTCCTGAACC | CCCTGTACTTATCCAGGCAGA |
| ***Gpx5*** | TCTAGCCAGCTATGTGCAGAC | TCCTTCCCATTAAGAGACAGAGC |
| ***Gpx7*** | TTCTGGAAGTACCTAGTGGACC | GCTCTGTAATACGGGGCTTGA |
| ***Gpx8*** | CCTTTCGCTGCCTACCCATTA | GAGTAGAAGCTGTTGGTTCTC |
| ***Pparg*** | CTCCAAGAATACCAAAGTGCGA | GCCTGATGCTTTATCCCCACA |
| ***Cebpa*** | AGTCGGTGGACAAGAACAGC | TCACTGGTCAACTCCAGCA |
| ***Gli1*** | TACCATGAGCCCTTCTTTAGGA | GCATCATTGAACCCCGAGTAG |
| ***Igf1*** | CTGGACCAGAGACCCTTTGC | GGACGGGGACTTCTGAGTCTT |
| ***Igf2*** | CCGTGGGCAAGTTCTTCCAATATG | ACGATGACGTTTGGCCTCTCTGAA |
| ***Ptch1*** | GCCTTGGCTGTGGGATTAAAG | CTTCTCCTATCTTCTGACGGGT |
| ***Fgf2*** | GCGACCCACACGTCAAACTA | TCCCTTGATAGACACAACTCCTC |
| ***Nrf2*** | CTTTAGTCAGCGACAGAAGGAC | AGGCATCTTGTTTGGGAATGTG |
| ***Sod1*** | AACCAGTTGTGTTGTCAGGAC | CCACCATGTTTCTTAGAGTGAGG |
| ***Sod2*** | CAGACCTGCCTTACGACTATGG | CTCGGTGGCGTTGAGATTGTT |
| ***Foxo1*** | CCCAGGCCGGAGTTTAACC | GTTGCTCATAAAGTCGGTGCT |
| ***Hmox1*** | AAGCCGAGAATGCTGAGTTCA | GCCGTGTAGATATGGTACAAGGA |
| ***Prdx1*** | AATGCAAAAATTGGGTATCCTGC | CGTGGGACACACAAAAGTAAAGT |
|  |  |  |
| **HUMAN** | **F** | **R** |
| ***ACTB*** | TCACTATTGGCAACGAGCG | AGGTCTTTACGGATGTCAACG |
| ***GPX7*** | CCTGCCTTCAAGTACCTGGC | TCTTCTCGCTTCAGTAGGATGAG |
| ***IBSP*** | CCCCACCTTTTGGGAAAACCA | TCCCCGTTCTCACTTTCATAGAT |
| ***PTH1R*** | AGTGCGAAAAACGGCTCAAG | GATGCCTTATCTTTCCTGGGC |
| ***BGLAP*** | CAGCGAGGTAGTGAAGAGAC | TGAAAGCCGATGTGGTCAG |
| ***COL1*** | GAGGGCCAAGACGAAGACATC | CAGATCACGTCATCGCACAAC |
| ***ALPL*** | CTATCCTGGCTCCGTGCTCC | GTTAACTGATGTTCCAATCCTGC |
| ***RUNX2*** | CCTTTACTTACACCCCGCCA | GGATCCTGACGAAGTGCCAT |
| ***PPARG*** | GGGATCAGCTCCGTGGATCT | TGCACTTTGGTACTCTTGAAGTT |
| ***CEBPA*** | AAACAACGCAACGTGGAGA | GCGGTCATTGTCACTGGTC |
| ***GPX1*** | CAGTCGGTGTATGCCTTCTCG | GAGGGACGCCACATTCTCG |
| ***GPX2*** | GGTAGATTTCAATACGTTCCGGG | TGACAGTTCTCCTGATGTCCAAA |
| ***GPX3*** | AGAGCCGGGGACAAGAGAA | ATTTGCCAGCATACTGCTTGA |
| ***GPX4*** | GAGGCAAGACCGAAGTAAACTAC | CCGAACTGGTTACACGGGAA |
| ***GPX8*** | TACTTAGGGCTGAAGGAACTGC | GGCTCCGATTCTCCAAACTGA |

Note

F, forward; R, reverse
